# CT angiography and CT perfusion improve prediction of infarct volume in patients with anterior circulation stroke

**DOI:** 10.1007/s00234-015-1636-z

**Published:** 2016-01-14

**Authors:** Tom van Seeters, Geert Jan Biessels, L. Jaap Kappelle, Irene C. van der Schaaf, Jan Willem Dankbaar, Alexander D. Horsch, Joris M. Niesten, Merel J. A. Luitse, Charles B. L. M. Majoie, Jan Albert Vos, Wouter J. Schonewille, Marianne A. A. van Walderveen, Marieke J. H. Wermer, Lucien E. M. Duijm, Koos Keizer, Joseph C. J. Bot, Marieke C. Visser, Aad van der Lugt, Diederik W. J. Dippel, F. Oskar H. W. Kesselring, Jeannette Hofmeijer, Geert J. Lycklama à Nijeholt, Jelis Boiten, Willem Jan van Rooij, Paul L. M. de Kort, Yvo B. W. E. M. Roos, Frederick J. A. Meijer, C. Constantijn Pleiter, Willem P. T. M. Mali, Yolanda van der Graaf, Birgitta K. Velthuis, on behalf of the Dutch acute stroke study (DUST) investigators

**Affiliations:** Department of Radiology, University Medical Center Utrecht, Heidelberglaan 100, HP E01.132 3584 CX Utrecht, The Netherlands; Department of Neurology, Brain Center Rudolf Magnus, University Medical Center Utrecht, Utrecht, The Netherlands; Department of Radiology, Academic Medical Center, Amsterdam, The Netherlands; Department of Radiology, St. Antonius Hospital, Nieuwegein, The Netherlands; Department of Neurology, St. Antonius Hospital, Nieuwegein, The Netherlands; Department of Radiology, Leiden University Medical Center, Leiden, The Netherlands; Department of Neurology, Leiden University Medical Center, Leiden, The Netherlands; Department of Radiology, Catharina Hospital, Eindhoven, The Netherlands; Department of Neurology, Catharina Hospital, Eindhoven, The Netherlands; Department of Radiology, VU University Medical Center, Amsterdam, The Netherlands; Department of Neurology, VU University Medical Center, Amsterdam, The Netherlands; Department of Radiology, Erasmus MC University Medical Center, Rotterdam, The Netherlands; Department of Neurology, Erasmus MC University Medical Center, Rotterdam, The Netherlands; Department of Radiology, Rijnstate Hospital, Arnhem, The Netherlands; Department of Neurology, Rijnstate Hospital, Arnhem, The Netherlands; Department of Radiology, Medical Center Haaglanden, The Hague, The Netherlands; Department of Neurology, Medical Center Haaglanden, The Hague, The Netherlands; Department of Radiology, St. Elisabeth Hospital, Tilburg, The Netherlands; Department of Neurology, St. Elisabeth Hospital, Tilburg, The Netherlands; Department of Neurology, Academic Medical Center, Amsterdam, The Netherlands; Department of Radiology, Radboud University Medical Center, Nijmegen, The Netherlands; Department of Radiology, St. Franciscus Hospital, Rotterdam, The Netherlands; Julius Center for Health Sciences and Primary Care, University Medical Center Utrecht, Utrecht, The Netherlands

**Keywords:** Ischemic stroke, Prediction, CT angiography, CT perfusion, Infarct volume

## Abstract

**Introduction:**

We investigated whether baseline CT angiography (CTA) and CT perfusion (CTP) in acute ischemic stroke could improve prediction of infarct presence and infarct volume on follow-up imaging.

**Methods:**

We analyzed 906 patients with suspected anterior circulation stroke from the prospective multicenter Dutch acute stroke study (DUST). All patients underwent baseline non-contrast CT, CTA, and CTP and follow-up non-contrast CT/MRI after 3 days. Multivariable regression models were developed including patient characteristics and non-contrast CT, and subsequently, CTA and CTP measures were added. The increase in area under the curve (AUC) and *R*^2^ was assessed to determine the additional value of CTA and CTP.

**Results:**

At follow-up, 612 patients (67.5 %) had a detectable infarct on CT/MRI; median infarct volume was 14.8 mL (interquartile range (IQR) 2.8–69.6). Regarding infarct presence, the AUC of 0.82 (95 % confidence interval (CI) 0.79–0.85) for patient characteristics and non-contrast CT was improved with addition of CTA measures (AUC 0.85 (95 % CI 0.82–0.87); *p* < 0.001) and was even higher after addition of CTP measures (AUC 0.89 (95 % CI 0.87–0.91); *p* < 0.001) and combined CTA/CTP measures (AUC 0.89 (95 % CI 0.87–0.91); *p* < 0.001). For infarct volume, adding combined CTA/CTP measures (*R*^2^ = 0.58) was superior to patient characteristics and non-contrast CT alone (*R*^2^ = 0.44) and to addition of CTA alone (*R*^2^ = 0.55) or CTP alone (*R*^2^ = 0.54; all *p* < 0.001).

**Conclusion:**

In the acute stage, CTA and CTP have additional value over patient characteristics and non-contrast CT for predicting infarct presence and infarct volume on follow-up imaging. These findings could be applied for patient selection in future trials on ischemic stroke treatment.

**Electronic supplementary material:**

The online version of this article (doi:10.1007/s00234-015-1636-z) contains supplementary material, which is available to authorized users.

## Introduction

Ischemic stroke is a major cause of death and disability worldwide [1]. In patients with clinical features of acute ischemic stroke, the underlying cause should be identified and different treatment options should be weighed in order to start optimal treatment as quickly as possible. Patient-specific information on expected infarct volume could improve the choice of acute therapy, as infarct volume is a frequently used outcome measure in intervention trials [2, 3] and is associated with clinical outcome [4–7].

CT angiography (CTA) and CT perfusion (CTP) can provide important diagnostic, etiologic, and also prognostic information in patients with acute ischemic stroke. CTA offers the possibility to determine the presence of an intracranial occlusion, to assess the leptomeningeal collateral circulation, and to visualize the endovascular access through the cervical arteries [8–12]. CTP is used to obtain measures of brain perfusion and to differentiate reversible ischemia (penumbra) from the irreversibly damaged infarct core [11–16]. In a previous study, we showed that CTA and CTP measures were strong predictors of clinical outcome [17], though in multivariable prediction models, their prognostic value in addition to easier-to-obtain measures, i.e., patient characteristics and non-contrast CT (NCCT), was limited. However, it is unclear whether CTA and CTP measures can help to predict both presence of an infarct and infarct volume on follow-up imaging.

The aim of the present study was to investigate whether baseline CTA and CTP measures in acute ischemic stroke patients can improve prediction of infarct presence and infarct volume on follow-up imaging when added to baseline patient characteristics and NCCT.

## Methods

### Study population

All patients participated in the Dutch acute stroke study (DUST), a prospective observational cohort study in six university and eight non-university hospitals in The Netherlands. A detailed description of the DUST study protocol has been published previously [18]. The DUST study population consists of patients (*n* = 1476) with symptoms of acute ischemic stroke of less than 9-h duration, who were enrolled between May 2009 and August 2013. Patients with another diagnosis than probable ischemic stroke on admission NCCT were excluded. All patients underwent NCCT, CTA, and CTP on admission and follow-up NCCT if possible. Ethical approval was obtained from the medical ethics committee of the University Medical Center Utrecht, The Netherlands, in addition to local approval from all participating hospitals. Informed consent was obtained from patients or their legal representative. The medical ethics committee waived the need for informed consent for patients who died before informed consent could be obtained.

For the present study, we selected patients who had a clinical suspicion of anterior circulation stroke at admission. This was determined by a neurologist in the acute stage and was defined as either total anterior circulation syndrome (TACS), partial anterior circulation syndrome (PACS), or lacunar syndrome (LACS) [19]. Additional exclusion criteria for the present study were absence of follow-up imaging and time between admission and follow-up imaging <12 h or >14 days.

### Candidate predictors

Candidate predictors were divided in patient characteristics, NCCT predictors, CTA predictors, and CTP predictors. Admission scans were assessed by one of three observers with at least 5 years of experience in neurovascular imaging, blinded for all clinical information except for the side of symptoms.

#### Patient characteristics and NCCT predictors

Patient characteristics were collected at baseline and included age, stroke severity determined by the National Institutes of Health Stroke Scale (NIHSS) [20], time between symptom onset and imaging, blood glucose level (mmol/L), and information on treatment with intravenous thrombolysis with recombinant tissue-type plasminogen activator (IV-rtPA), intra-arterial thrombolysis, or mechanical thrombectomy [21–24]. On NCCT, the presence of a hyperdense vessel sign was recorded and early ischemic changes were assessed with the Alberta Stroke Program Early CT Score (ASPECTS) [25–28].

#### CTA predictors

CTA measures were ASPECTS on CTA source images, a proximal intracranial arterial occlusion (either distal internal carotid artery or M1 segment of the middle cerebral artery), poor leptomeningeal collaterals (≤50 % collateral filling of the affected territory), and >70 % stenosis or occlusion of the internal carotid artery ipsilateral to the affected hemisphere [8–11, 28–32].

#### CTP predictors

CTP measures were ASPECTS on cerebral blood volume (CBV) and mean transit time (MTT) maps, penumbra area (cm^2^), and infarct core area (cm^2^) [15, 28, 33, 34]. Penumbra and infarct core areas were calculated using previously reported MTT and CBV thresholds [34]. We accounted for differences in CTP coverage by using the sum of penumbra and infarct core areas on the two ASPECTS levels, as CTP coverage included those levels in all patients.

### Study outcome

The first study outcome was presence of infarct on follow-up imaging. The default follow-up imaging modality was NCCT after 3 days or at the time of clinical deterioration or earlier discharge. Follow-up MRI was used if this had been performed for clinical reasons instead of NCCT. The second study outcome was infarct volume (in mL). This was obtained by manually delineating the hypodense infarcted area(s) on axial NCCT slices and hyper-intense area(s) on axial DWI slices on MRI. The surface of these area(s) was subsequently multiplied by the slice thickness to obtain the infarct volume. Observers were blinded for admission CTA and CTP when they delineated the infarcts. Clinical outcome was assessed at 90 days using the modified Rankin Scale (mRS) [35].

### Analyses

#### Univariable analyses

Logistic regression was used to determine the relation between each of the patient characteristics and CT predictors and presence of infarct on follow-up imaging. This was expressed as an odds ratio with 95 % confidence interval (CI). We also calculated the positive predictive value (PPV) for all predictors, indicating the probability of infarct presence if a predictor was abnormal. Single imputation was performed to account for missing data. Continuous predictors were truncated at the first and 99th percentile to minimize the effect of outliers [36].

#### Multivariable analyses

To investigate whether CTA and CTP would improve the prognostic value of patient characteristics and NCCT predictors, four different multivariable logistic regression models were fitted to predict infarct presence on follow-up imaging. The first model contained patient characteristics and NCCT (model 1). In the subsequent two models, either CTA measures (model 2a) or CTP measures (model 2b) were added to the first model. In the final model, both CTA and CTP measures were added to the first model (model 3). Shrinkage of the model coefficients was performed to correct for optimism, and the optimal shrinkage factor was determined by bootstrap resampling with 1000 bootstrap samples [36]. Performance of the models was assessed with receiver operator characteristic (ROC) analyses and corresponding area under the curve (AUC) values, which indicate the ability to differentiate between patients with and without an infarct on follow-up imaging. Differences in AUC were tested for statistical significance [37].

To assess the additional value of CTA and CTP for prediction of infarct volume, we used Tobit (censored) regression analyses [38, 39]. Tobit regression is useful for situations where the dependent variable is either 0 or above (but not below), as is the case for infarct volume in our study. In one single step, it determines both the probability of infarct volume being above 0 mL and changes in infarct volume when it is above 0 mL [40]. The results of the Tobit regression analyses are expressed as beta coefficients with 95 % CI. Differences between the Tobit models were determined with likelihood ratio tests. We explored the possibility of analyzing our data with linear regression. However, the conditions for linear regression were not fulfilled as the residuals were not normally distributed and residual regression plots suggested that there was no homoscedasticity. We then performed analyses after transformation of the infarct volume including natural logarithm, square, cube, square root, cube root, and reciprocal transformations. As the conditions for linear regression were also not fulfilled after these transformations, we considered linear regression not suitable for our data.

To assess whether MRI assessment instead of NCCT would affect our findings, we repeated the analyses after excluding patients with MRI as follow-up modality.

Finally, we determined whether infarct volume on follow-up imaging was predictive for clinical outcome. Statistical significance was tested with the Kruskal-Wallis test. All analyses were performed with R version 3.0.2.

## Results

**Fig. 1:**
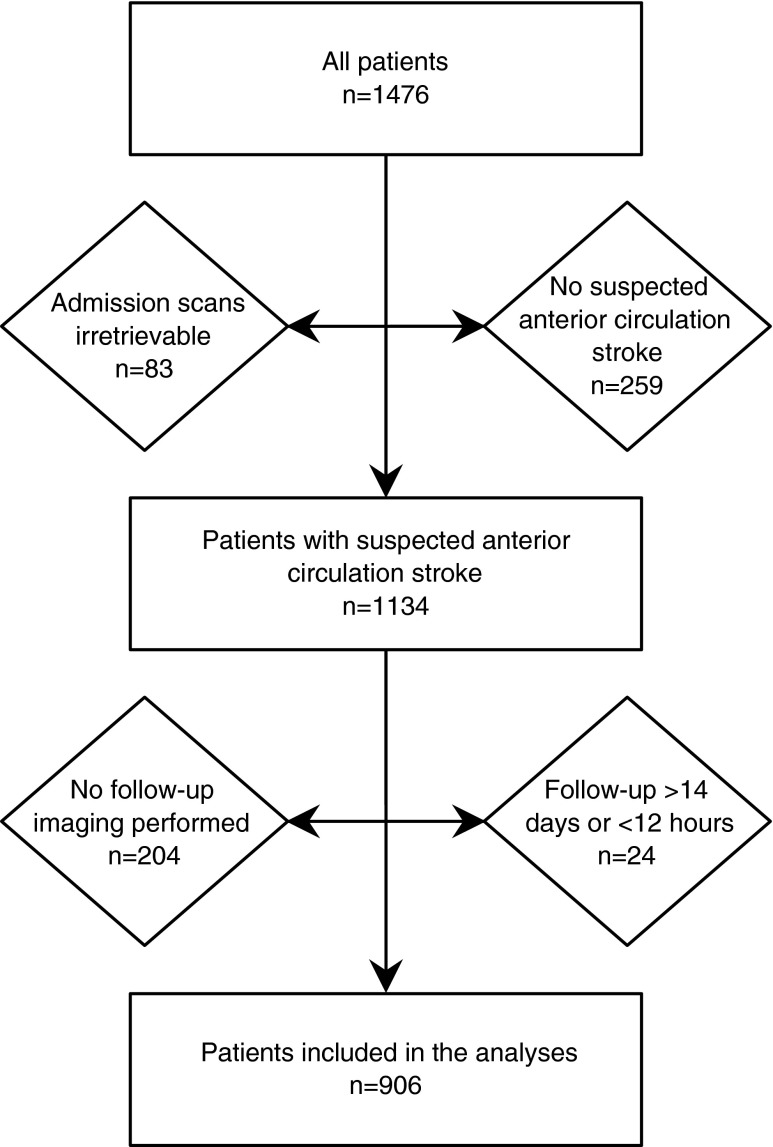
Flowchart depicting the number of patients included in the study and remaining for the analyses

After applying the inclusion and exclusion criteria, 906 patients remained for the analyses (Fig. **1**). The mean age was 67.4 ± 13.8 years, 527 patients (58.2 %) were male, and the median NIHSS was 7 (interquartile range (IQR) 4–13). IV-rtPA was given to 579 patients (63.9 %), and 60 patients (6.6 %) received intra-arterial treatment. Twenty-one patients (2.3 %) received intra-arterial treatment without prior IV-rtPA. The median interval between admission and follow-up imaging was 2.9 days (IQR 1.9–3.7 days). Follow-up imaging was performed with NCCT in 839 patients (92.6 %) and MRI in 67 patients (7.4 %). An infarct was detected in 612 patients (67.5 %) on follow-up imaging, with a median infarct volume of 14.8 mL (IQR 2.8–69.6). Infarct volumes were higher on follow-up NCCT (15.6 mL (IQR 3.1–73.8)) than on follow-up MRI (4.0 mL (IQR 1.0–26.8); *p* = 0.001). Fifty-six patients (6.2 %) had a posterior circulation infarct on follow-up imaging, indicating clinical misclassification of suspected infarct location at baseline. Additional patient characteristics can be found in Table 1.

**Table 1:** Patient characteristics

	All patients	No infarct on follow-up	Infarct volume <14.8 mL^a^	Infarct volume ≥14.8 mL^a^
Number of patients	906 (100.0)	294 (32.5)	306 (33.8)	306 (33.8)
Clinical measures				
Age (years)	67.4 (13.8)	69.2 (13.3)	68.3 (13.5)	64.9 (14.2)
Male gender	527 (58.2)	154 (52.4)	178 (58.2)	195 (63.7)
Stroke severity (NIHSS)	7 (4–13)	4 (3–6)	6 (3–10)	13 (8–17)
Time from symptom onset to scan (minutes)	113 (72–180)	116 (74–172)	121 (75–197)	101 (67–170)
IV-rtPA	579 (63.9)	188 (63.9)	186 (60.8)	205 (67.0)
Intra-arterial thrombolysis or mechanical thrombectomy	60 (6.6)	3 (1.0)	17 (5.6)	40 (13.1)
Smoking	251 (29.6)	78 (28.3)	90 (30.9)	83 (29.6)
Glucose (mmol/L)	6.5 (5.8–7.8)	6.3 (5.6–7.3)	6.5 (5.7–7.8)	6.8 (6.1–8.3)
Systolic blood pressure (mmHg)	157 (29.0)	160 (29.2)	159 (30.4)	153 (26.8)
Diastolic blood pressure (mmHg)	85 (16.9)	86 (16.3)	87 (17.4)	84 (17.0)
Clinical stroke subtype				
Total anterior circulation syndrome (TACS)	216 (23.8)	22 (7.5)	49 (16.0)	145 (47.4)
Partial anterior circulation syndrome (PACS)	539 (59.5)	184 (62.6)	202 (66.0)	153 (50.0)
Lacunar syndrome (LACS)	151 (16.7)	88 (29.9)	55 (18.0)	8 (2.6)
Non-contrast CT findings				
Hyperdense vessel sign	204 (22.5)	7 (2.4)	46 (15.0)	151 (49.5)
Non-contrast CT ASPECTS	10 (10–10)	10 (10–10)	10 (10–10)	10 (7–10)
CT angiography findings				
CT angiography source images ASPECTS	10 (8–10)	10 (10–10)	10 (9–10)	7 (5–10)
Proximal intracranial occlusion	255 (28.6)	12 (4.1)	70 (23.5)	173 (57.1)
Poor collaterals	122 (13.7)	4 (1.4)	18 (6.1)	100 (33.1)
Significant ipsilateral carotid stenosis or occlusion	156 (17.5)	19 (6.6)	43 (14.4)	94 (31.0)
CT perfusion findings				
Cerebral blood volume (CBV) ASPECTS	10 (7–10)	10 (10–10)	10 (9–10)	7 (5–8)
Mean transit time (MTT) ASPECTS	8 (4–10)	10 (10–10)	8 (5–10)	3 (1–6)
Penumbra area (cm^2^)^b^	23.0 (9.0–41.7)	15.3 (4.2–36.3)	18.8 (6.2–34.8)	26.9 (12.3–45.2)
Infarct core area (cm^2^)^b^	6.7 (1.5–21.0)	1.1 (0.0–5.0)	3.1 (0.4–6.9)	17.0 (5.2–32.5)
Clinical outcome				
Poor outcome at 90 days (mRS 3–6)	344 (38.4)	65 (22.6)	94 (30.9)	185 (60.7)
Follow-up imaging				
Infarct volume (mL)	3.0 (0.0–36.5)	0.0 (0.0–0.0)	2.8 (1.1–6.5)	69.6 (34.3–152.1)

All data are displayed as mean (standard deviation), median (interquartile range), or *n* (%).

^a^Median split for infarct volume in patients with an infarct on follow-up imaging

^b^In patients with a perfusion deficit

### Prediction of infarct presence on follow-up imaging

Univariable analyses showed a strong relation between all abnormal imaging measures at baseline and the presence of an infarct on follow-up imaging (Table 2). For a random patient in our study, the probability of having an infarct on follow-up imaging was 67.5 %, not taking any patient characteristics or imaging findings into account. However, if one or more imaging findings were abnormal, the probability that an infarct was present increased to 85–100 %.

**Table 2:** Univariable analyses for prediction of infarct presence on follow-up imaging (*n* = 906)

Predictor	OR	95 % confidence interval	PPV^a^ (%)
Age (per decade)	0.87	0.78–0.97**	
Lowest tertile (<62.3 years)			71
Middle tertile (62.3–74.0 years)			71
Highest tertile (≥74.0 years)			61
Stroke severity (NIHSS)			
NIHSS 1–2	1.00 (ref)		46
NIHSS 3–4	1.17	0.77–1.78	50
NIHSS 5–7	2.18	1.47–3.24***	65
NIHSS 8–13	6.94	4.14–11.64***	86
NIHSS >13	22.04	10.70–45.39***	95
Time from symptom onset to scan (per hour)	1.04	0.97–1.12	
Lowest tertile (≤86 min)			69
Middle tertile (86–147 min)			67
Highest tertile (≥147 min)			66
Admission glucose level (per mmol/L)	1.08	1.01–1.15*	
Lowest tertile (≤6.0 mmol/L)			58
Middle tertile (6.0–7.2 mmol/L)			71
Highest tertile (≥7.2 mmol/L)			74
IV-rtPA, intra-arterial thrombolysis, or mechanical thrombectomy	1.16	0.87–1.56	69
Non-contrast CT predictors			
Hyperdense vessel sign	19.61	9.09–42.29***	97
Non-contrast CT ASPECTS (per point decrease)	9.77	4.21–22.67***	
ASPECTS 10			59
ASPECTS 8–9			96
ASPECTS ≤7			100
CT angiography predictors			
CT angiography source images ASPECTS (per point decrease)	3.47	2.48–4.86***	
ASPECTS 10			53
ASPECTS 7–9			96
ASPECTS ≤6			98
Proximal intracranial occlusion	15.90	8.73–28.97***	95
Poor collaterals	17.68	6.46–48.39***	97
Significant ipsilateral carotid stenosis or occlusion	3.99	2.44–6.52***	87
CT perfusion predictors			
Cerebral blood volume (CBV) ASPECTS (per point decrease)	4.83	3.43–6.79***	
ASPECTS 10			44
ASPECTS 7–9			95
ASPECTS ≤6			99
Mean transit time (MTT) ASPECTS (per point decrease)	1.77	1.61–1.95***	
ASPECTS 10			29
ASPECTS 6–9			85
ASPECTS ≤5			94
Penumbra area (per SD; 19.9 cm^2^)	6.37	4.45–9.11***	
0.0 cm^2^			34
0.0–18.1 cm^2^			89
>18.1 cm^2^			94
Infarct core area (per SD; 13.7 cm^2^)	38.86	16.18–93.34***	
0.0 cm^2^			38
0.0–5.4 cm^2^			87
≥5.4 cm^2^			96

*p < 0.05; **p < 0.01; ***p < 0.001

^a^Prior probability of infarct presence is 67.5 %

For the multivariable analyses, the following bootstrap-derived shrinkage factors were applied to the model coefficients: 0.92 for model 1, 0.88 for model 2a, 0.90 for model 2b, and 0.84 for model 3. Model descriptions, coefficients, odds ratios, and AUC values are presented in Online Table 1.

The basic prognostic model including patient characteristics and NCCT (model 1) had a high predictive value for infarct presence, indicated by an AUC value of 0.82 (95 % CI 0.79–0.85). Stroke severity (NIHSS), presence of a hyperdense vessel sign, and ASPECTS on NCCT had a strong predictive value for infarct presence in this model (Online Table 1). Addition of CTA measures to the basic model (model 2a) improved the predictive value, as shown by the AUC value of 0.85 (95 % CI 0.82–0.87; *p* < 0.001). In this model, ASPECTS on CTA source images and presence of a proximal intracranial occlusion were the strongest CTA predictors of infarct presence on follow-up imaging. Addition of CTP measures alone (model 2b; AUC 0.89 (95 % CI (0.87–0.91)) or in combination with CTA measures (model 3; AUC 0.89 (95 % CI (0.87–0.91)) also improved the prognostic value when they were added to the basic model (both *p* < 0.001) and was superior to addition of CTA measures alone (both *p* < 0.001). For CTP, penumbra area and ASPECTS on CBV maps were independent predictors of infarct presence (Online Table 1). Addition of combined CTA and CTP measures was not superior to addition of CTP measures alone (*p* = 0.19). Results were comparable when patients with follow-up MRI instead of NCCT were excluded from the analyses and when patients who had a posterior circulation infarct on follow-up imaging were excluded.

**Fig. 2:**
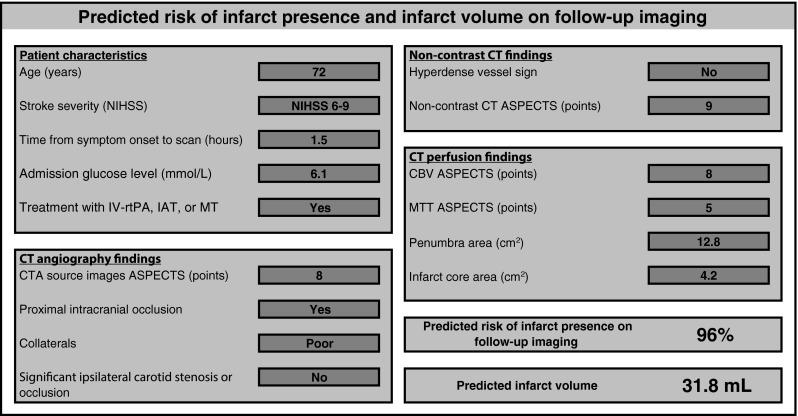
Example of a predicted risk of infarct presence and predicted infarct volume for an individual patient using an interactive calculation sheet

We used the multivariable models to calculate the predicted risk of infarct presence on follow-up imaging and divided patients into tertiles of low, intermediate, and high predicted risk. Next, we calculated for each tertile the actual proportion of patients that had an infarct on follow-up imaging. As can be seen in Table 3, the contrast between the low- and high-risk tertiles was largest for the model including both CTA and CTP measures: 28 versus 99 % presence of infarct at follow-up, respectively. An example of an interactive calculation sheet to make predictions for infarct presence and infarct volume for individual patients is provided in Fig. **2** (see Online Table 3 for the interactive calculation sheet).

**Table 3:** Actual risk of infarct presence on follow-up imaging according to tertiles of predicted risk for the model with patient characteristics and non-contrast CT (model 1) and with additional CT angiography (model 2a), CT perfusion (model 2b), and combined CT angiography and CT perfusion measures (model 3)

	Infarct on follow-up/n	Percentage (%)
All patients	612/906	68
Model 1—patient characteristics and non-contrast CT		
Lowest predicted risk tertile	119/302	39
Intermediate predicted risk tertile	200/302	66
Highest predicted risk tertile	293/302	97
Model 2a—addition of CT angiography		
Lowest predicted risk tertile	107/302	35
Intermediate predicted risk tertile	210/302	70
Highest predicted risk tertile	295/302	98
Model 2b—addition of CT perfusion		
Lowest predicted risk tertile	86/302	28
Intermediate predicted risk tertile	229/302	76
Highest predicted risk tertile	297/302	98
Model 3—addition of CT angiography and CT perfusion		
Lowest predicted risk tertile	86/302	28
Intermediate predicted risk tertile	228/302	75
Highest predicted risk tertile	298/302	99

### Prediction of infarct volume

CTA and CTP improved the prediction of infarct volume when they were added to patient characteristics and NCCT (Online Table 2). The models with addition of either CTA measures (model 2a; *R*^2^ = 0.55) or CTP measures (model 2b; *R*^2^ = 0.54) were superior to the model with patient characteristics and NCCT (model 1; *R*^2^ = 0.44; both *p* < 0.001). Furthermore, addition of combined CTA and CTP measures (model 3; *R*^2^ = 0.58) was superior to addition of CTA or CTP alone (both *p* < 0.001). In the model including both CTA and CTP measures, independent predictors of infarct volume were ASPECTS on NCCT, CTA source images, and CBV maps, poor collaterals, ipsilateral ICA stenosis or occlusion, and infarct core area. Results were comparable if patients with follow-up MRI and patients with a posterior circulation infarct on follow-up imaging were excluded from the analyses.

### Infarct volume and clinical outcome

**Fig. 3:**
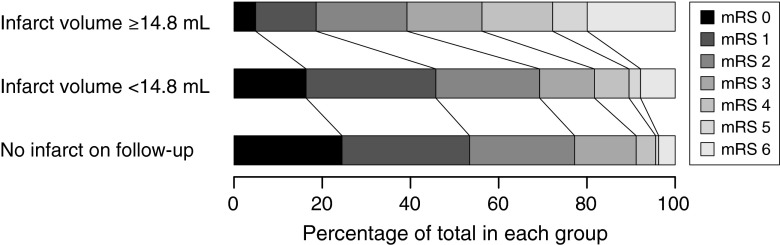
Infarct volume and clinical outcome. The range of mRS scores is depicted within patients with a large infarct, small infarct, or no infarct on follow-up imaging. Patients with an infarct on follow-up imaging were dichotomized at the median infarct volume (14.8 mL)

Patients with larger infarct volumes on follow-up imaging had higher mRS scores at 90 days than patients with smaller infarct volumes, while patients without a visible infarct on follow-up imaging had the lowest mRS scores (Fig. **3**; *p* < 0.001).

## Discussion

Our study shows that CTA and CTP have additional value over patient characteristics and NCCT for predicting infarct presence and infarct volume on follow-up imaging.

No other large prospective study has determined the additional value of CTA or CTP measures for prediction of infarct presence and infarct volume on follow-up imaging, although several studies investigated the prognostic value of individual NCCT, CTA, or CTP measures. However, these studies mostly included preselected patient populations such as patients fulfilling criteria for IV-rtPA [15], patients treated with intra-arterial thrombolysis [41, 42], patients with a confirmed occlusion [30, 42], patients with recanalization [42], or patients with a confirmed ischemic stroke [14, 29, 30, 42]. The patients in our study represent a cohort of all patients with a suspected acute ischemic stroke in the anterior circulation. This means that the patients in our study received different forms of treatment including IV-rtPA, intra-arterial thrombolysis, or mechanical thrombectomy or none of these treatment options. By using an unselected anterior circulation stroke population and adding treatment as a covariate to the analyses, our results are likely to be more generalizable to a broader stroke population. We restricted our study population to patients with a suspected anterior circulation stroke, because CTP thresholds to determine penumbra and infarct core have not been validated for posterior circulation stroke and also because we expected that the relation between imaging measures and infarct volume would be different for patients with anterior and posterior circulation stroke. Nonetheless, 6 % of the patients in our study had an infarct in the posterior circulation on follow-up imaging. This percentage is consistent with previous literature and probably reflects difficulties in infarct localization based on clinical information alone [43, 44]. However, it could have led to a small underestimation of the coefficients that we have found—especially for the imaging measures—as they are more specifically focused on the anterior circulation than patient characteristics.

Regarding the prognostic value of individual CTP and CTA measures, previous studies were consistent with our results and identified ASPECTS on NCCT [8, 9, 29, 30], CTA source images [8, 9, 30, 42], CBV maps [15, 42], and MTT maps [15] as predictors of infarct volume. Other studies did not use ASPECTS for assessment of CTP, but instead used CBV volume at baseline to be a predictor of infarct volume [14, 29]. The predictive value of collateral status for infarct volume is also consistent with previous research [41]. A significant stenosis or occlusion of the internal carotid artery, ipsilateral to the suspected hemisphere, predicted a larger infarct volume in our study, but this was not examined in previous studies. The larger infarct volume in these patients may result from failure of vasodilatative cerebral autoregulatory mechanisms in patients with severe stenosis and subsequent chronic hypoxic stress [45]. Successful recanalization is known to result in a smaller final infarct volume [46]. However, we did not include this information in our prediction models, as we aimed to use only information that is available upon admission to the hospital. In this way, our models can be used to inform neurologists and patients in the very acute stage.

In a previous study, we showed strong predictive values of individual abnormal imaging measures for prediction of functional outcome after 90 days in univariable analyses [17]. However, using the same type of modeling as we performed in the present study, there was no additional value of CTA or CTP measures when they were added to clinical features and NCCT. The differences between these two studies can be explained by infarct location [47] and by factors other than admission imaging findings that can also determine the clinical outcome after 90 days, including occurrence of another infarct [48] and post-stroke infections [49].

We acknowledge some limitations to our study. Infarct size was measured on either CT or MRI. As the default follow-up modality was NCCT, it is possible that some smaller infarcts were not detected. Median infarct volumes were larger on follow-up NCCT than on follow-up MRI, which can be explained by the fact that MRI was performed instead of NCCT when there was a strong suspicion of an infarct, but admission CT did not show any abnormalities. However, as the results were similar when we repeated the analyses without patients with MRI as follow-up modality, it is unlikely that this has caused a major bias. In addition, infarct volume was measured after 3 days which could have led to an overestimation of the true infarct volume due to the presence of cytotoxic edema, as it has been shown that infarct volume is smaller after 3 months [50]. Infarct volumes on MRI were measured on DWI, which is reliable but less accurate when compared with FLAIR lesions after 30 days [51]. Furthermore, CTP coverage did not include the entire brain. Finally, the regression coefficients of the Tobit analyses are applicable to the (theoretical) uncensored infarct volume values, while in practice, this variable is censored. Nonetheless, we think that Tobit regression is still the most appropriate method to analyze our data.

## Conclusions

This study showed that adding CTA and CTP measures to patient characteristics and NCCT improves prediction of infarct presence and infarct volume on follow-up imaging. CTA and CTP help the clinician to predict which patients with acute anterior circulation stroke symptoms actually develop an infarct and to predict the infarct volume. These results could be used for patient selection in future trials on treatment of acute ischemic stroke.

## Electronic supplementary material

Below is the link to the electronic supplementary material.

ESM 1 (PDF 138 kb)

ESM 2 (XLSX 28 kb)

## References

[1] Go AS, Mozaffarian D, Roger VL, Benjamin EJ, Berry JD, Borden WB, Bravata DM, Dai S, Ford ES, Fox CS, Franco S, Fullerton HJ, Gillespie C, Hailpern SM, Heit JA, Howard VJ, Huffman MD, Kissela BM, Kittner SJ, Lackland DT, Lichtman JH, Lisabeth LD, Magid D, Marcus GM, Marelli A, Matchar DB, McGuire DK, Mohler ER, Moy CS, Mussolino ME, Nichol G, Paynter NP, Schreiner PJ, Sorlie PD, Stein J, Turan TN, Virani SS, Wong ND, Woo D, Turner MB (2013). Heart disease and stroke statistics—2013 update: a report from the American Heart Association. Circulation.

[2] Berkhemer OA, Fransen PS, Beumer D, van den Berg LA, Lingsma HF, Yoo AJ, Schonewille WJ, Vos JA, Nederkoorn PJ, Wermer MJ, van Walderveen MA, Staals J, Hofmeijer J, van Oostayen JA, Lycklama A, Nijeholt GJ, Brouwer PA, Emmer BJ, de Bruijn SF, van Dijk LC, Kappelle LJ, Lo RH, van Dijk EJ, de Vries J, de Kort PL, van Rooij WJ, van den Berg JS, van Hasselt BA, Aerden LA, Dallinga RJ, Visser MC, Bot JC, Vroomen PC, Eshghi O, Schreuder TH, Heijboer RJ, Keizer K, Tielbeek AV, den Hertog HM, Gerrits DG, van den Berg-Vos RM, Karas GB, Steyerberg EW, Flach HZ, Marquering HA, Sprengers ME, Jenniskens SF, Beenen LF, van den Berg R, Koudstaal PJ, van Zwam WH, Roos YB, van der Lugt A, van Oostenbrugge RJ, Majoie CB, Dippel DW (2015). A randomized trial of intraarterial treatment for acute ischemic stroke. N Engl J Med.

[3] Jovin TG, Chamorro A, Cobo E, de Miquel MA, Molina CA, Rovira A, San Roman L, Serena J, Abilleira S, Ribo M, Millan M, Urra X, Cardona P, Lopez-Cancio E, Tomasello A, Castano C, Blasco J, Aja L, Dorado L, Quesada H, Rubiera M, Hernandez-Perez M, Goyal M, Demchuk AM, von Kummer R, Gallofre M, Davalos A (2015). Thrombectomy within 8 hours after symptom onset in ischemic stroke. N Engl J Med.

[4] De Reuck J, Van de Velde E, Van Maele G, Wissaert W (2003). The prognostic significance of changes in X-ray attenuation on CT in established cerebral infarcts. Cerebrovasc Dis.

[5] Johnston KC, Barrett KM, Ding YH, Wagner DP (2009). Clinical and imaging data at 5 days as a surrogate for 90-day outcome in ischemic stroke. Stroke.

[6] Yoo AJ, Chaudhry ZA, Nogueira RG, Lev MH, Schaefer PW, Schwamm LH, Hirsch JA, Gonzalez RG (2012). Infarct volume is a pivotal biomarker after intra-arterial stroke therapy. Stroke.

[7] Ribo M, Flores A, Mansilla E, Rubiera M, Tomasello A, Coscojuela P, Pagola J, Rodriguez-Luna D, Muchada M, Alvarez-Sabin J, Molina CA (2014). Age-adjusted infarct volume threshold for good outcome after endovascular treatment. J Neurointerv Surg.

[8] Coutts SB, Lev MH, Eliasziw M, Roccatagliata L, Hill MD, Schwamm LH, Pexman JH, Koroshetz WJ, Hudon ME, Buchan AM, Gonzalez RG, Demchuk AM (2004). ASPECTS on CTA source images versus unenhanced CT: added value in predicting final infarct extent and clinical outcome. Stroke.

[9] Camargo EC, Furie KL, Singhal AB, Roccatagliata L, Cunnane ME, Halpern EF, Harris GJ, Smith WS, Gonzalez RG, Koroshetz WJ, Lev MH (2007). Acute brain infarct: detection and delineation with CT angiographic source images versus nonenhanced CT scans. Radiology.

[10] Puetz V, Dzialowski I, Hill MD, Subramaniam S, Sylaja PN, Krol A, O’Reilly C, Hudon ME, Hu WY, Coutts SB, Barber PA, Watson T, Roy J, Demchuk AM (2008). Intracranial thrombus extent predicts clinical outcome, final infarct size and hemorrhagic transformation in ischemic stroke: the clot burden score. Int J Stroke.

[11] Tan JC, Dillon WP, Liu S, Adler F, Smith WS, Wintermark M (2007). Systematic comparison of perfusion-CT and CT-angiography in acute stroke patients. Ann Neurol.

[12] Schramm P, Schellinger PD, Klotz E, Kallenberg K, Fiebach JB, Kulkens S, Heiland S, Knauth M, Sartor K (2004). Comparison of perfusion computed tomography and computed tomography angiography source images with perfusion-weighted imaging and diffusion-weighted imaging in patients with acute stroke of less than 6 hours’ duration. Stroke.

[13] Schaefer PW, Barak ER, Kamalian S, Gharai LR, Schwamm L, Gonzalez RG, Lev MH (2008). Quantitative assessment of core/penumbra mismatch in acute stroke: CT and MR perfusion imaging are strongly correlated when sufficient brain volume is imaged. Stroke.

[14] Muir KW, Halbert HM, Baird TA, McCormick M, Teasdale E (2006). Visual evaluation of perfusion computed tomography in acute stroke accurately estimates infarct volume and tissue viability. J Neurol Neurosurg Psychiatry.

[15] Parsons MW, Pepper EM, Chan V, Siddique S, Rajaratnam S, Bateman GA, Levi CR (2005). Perfusion computed tomography: prediction of final infarct extent and stroke outcome. Ann Neurol.

[16] Wintermark M, Reichhart M, Cuisenaire O, Maeder P, Thiran JP, Schnyder P, Bogousslavsky J, Meuli R (2002). Comparison of admission perfusion computed tomography and qualitative diffusion- and perfusion-weighted magnetic resonance imaging in acute stroke patients. Stroke.

[17] van Seeters T, Biessels GJ, Kappelle LJ, van der Schaaf IC, Dankbaar JW, Horsch AD, Niesten JM, Luitse MJ, Majoie CB, Vos JA, Schonewille WJ, van Walderveen MA, Wermer MJ, Duijm LE, Keizer K, Bot JC, Visser MC, van der Lugt A, Dippel DW, Kesselring FO, Hofmeijer J, Lycklama ANGJ, Boiten J, van Rooij WJ, de Kort PL, Roos YB, van Dijk EJ, Pleiter CC, Mali WP, van der Graaf Y, Velthuis BK (2015) The prognostic value of CT angiography and CT perfusion in acute ischemic stroke. Cerebrovasc Dis 40:258–269 10.1159/000441088 26484857

[18] van Seeters T, Biessels GJ, van der Schaaf IC, Dankbaar JW, Horsch AD, Luitse MJ, Niesten JM, Mali WP, Kappelle LJ, van der Graaf Y, Velthuis BK (2014) Prediction of outcome in patients with suspected acute ischaemic stroke with CT perfusion and CT angiography: the Dutch acute stroke trial (DUST) study protocol. BMC Neurol 14:37 10.1186/1471-2377-14-37 PMC3939816 24568540

[19] Bamford J, Sandercock P, Dennis M, Burn J, Warlow C (1991). Classification and natural history of clinically identifiable subtypes of cerebral infarction. Lancet.

[20] Brott T, Adams HP, Olinger CP, Marler JR, Barsan WG, Biller J, Spilker J, Holleran R, Eberle R, Hertzberg V, Rorick M, Moomaw CJ, Walker M (1989). Measurements of acute cerebral infarction: a clinical examination scale. Stroke.

[21] Woo D, Broderick JP, Kothari RU, Lu M, Brott T, Lyden PD, Marler JR, Grotta JC (1999). Does the National Institutes of Health Stroke Scale favor left hemisphere strokes?. Stroke.

[22] Shimoyama T, Kimura K, Uemura J, Saji N, Shibazaki K (2014). Elevated glucose level adversely affects infarct volume growth and neurological deterioration in non-diabetic stroke patients, but not diabetic stroke patients. Eur J Neurol.

[23] Rangaraju S, Owada K, Noorian AR, Nogueira RG, Nahab F, Glenn BA, Belagaje SR, Anderson AM, Frankel MR, Gupta R (2013). Comparison of final infarct volumes in patients who received endovascular therapy or intravenous thrombolysis for acute intracranial large-vessel occlusions. JAMA Neurology.

[24] Baird TA, Parsons MW, Phan T, Butcher KS, Desmond PM, Tress BM, Colman PG, Chambers BR, Davis SM (2003). Persistent poststroke hyperglycemia is independently associated with infarct expansion and worse clinical outcome. Stroke.

[25] Barber PA, Demchuk AM, Zhang J, Buchan AM (2000). Validity and reliability of a quantitative computed tomography score in predicting outcome of hyperacute stroke before thrombolytic therapy. Lancet.

[26] Pexman JH, Barber PA, Hill MD, Sevick RJ, Demchuk AM, Hudon ME, Hu WY, Buchan AM (2001). Use of the Alberta Stroke Program Early CT Score (ASPECTS) for assessing CT scans in patients with acute stroke. AJNR Am J Neuroradiol.

[27] Puetz V, Dzialowski I, Hill MD, Demchuk AM (2009). The Alberta Stroke Program Early CT Score in clinical practice: what have we learned?. Int J Stroke.

[28] van Seeters T, Biessels GJ, Niesten JM, van der Schaaf IC, Dankbaar JW, Horsch AD, Mali WP, Kappelle LJ, van der Graaf Y, Velthuis BK (2013) Reliability of visual assessment of non-contrast CT, CT angiography source images and CT perfusion in patients with suspected ischemic stroke. PLoS ONE 8:e75615 10.1371/journal.pone.0075615 PMC3792960 24116061

[29] Tan IY, Demchuk AM, Hopyan J, Zhang L, Gladstone D, Wong K, Martin M, Symons SP, Fox AJ, Aviv RI (2009). CT angiography clot burden score and collateral score: correlation with clinical and radiologic outcomes in acute middle cerebral artery infarct. AJNR Am J Neuroradiol.

[30] Bhatia R, Bal SS, Shobha N, Menon BK, Tymchuk S, Puetz V, Dzialowski I, Coutts SB, Goyal M, Barber PA, Watson T, Smith EE, Demchuk AM (2011). CT Angiographic source images predict outcome and final infarct volume better than noncontrast CT in proximal vascular occlusions. Stroke.

[31] Rothwell PM, Eliasziw M, Gutnikov SA, Fox AJ, Taylor DW, Mayberg MR, Warlow CP, Barnett HJ (2003). Analysis of pooled data from the randomised controlled trials of endarterectomy for symptomatic carotid stenosis. Lancet.

[32] Aoki J, Tateishi Y, Cummings CL, Cheng-Ching E, Ruggieri P, Hussain MS, Uchino K (2014). Collateral flow and brain changes on computed tomography angiography predict infarct volume on early diffusion-weighted imaging. J Stroke Cerebrovasc Dis.

[33] Aviv RI, Mandelcorn J, Chakraborty S, Gladstone D, Malham S, Tomlinson G, Fox AJ, Symons S (2007). Alberta stroke program early ct scoring of ct perfusion in early stroke visualization and assessment. AJNR Am J Neuroradiol.

[34] Wintermark M, Flanders AE, Velthuis BK, Meuli R, van Leeuwen MS, Goldsher D, Pineda C, Serena J, van der Schaaf IC, Waaijer A, Anderson J, Nesbit G, Gabriely I, Medina V, Quiles A, Pohlman S, Quist M, Schnyder P, Bogousslavsky J, Dillon WP, Pedraza S (2006). Perfusion-CT assessment of infarct core and penumbra: receiver operating characteristic curve analysis in 130 patients suspected of acute hemispheric stroke. Stroke.

[35] van Swieten JC, Koudstaal PJ, Visser MC, Schouten HJ, van Gijn J (1988). Interobserver agreement for the assessment of handicap in stroke patients. Stroke.

[36] Steyerberg EW (2009). Clinical prediction models: a practical approach to development, validation, and updating.

[37] De Long ER, DeLong DM, Clarke-Pearson DL (1988). Comparing the areas under two or more correlated receiver operating characteristic curves: a nonparametric approach. Biometrics.

[38] Tobin J (1958). Estimation of relationships for limited dependent variables. Econometrica.

[39] UCLA: Statistical consulting group R data analysis examples: Tobit models. Available via http://www.ats.ucla.edu/stat/r/dae/tobit.htm. Accessed July 7 2015

[40] McDonald JF, Moffitt RA (1980). The uses of Tobit analysis. Rev Econ Stat.

[41] Angermaier A, Langner S, Kirsch M, Kessler C, Hosten N, Khaw AV (2011). CT-angiographic collateralization predicts final infarct volume after intra-arterial thrombolysis for acute anterior circulation ischemic stroke. Cerebrovasc Dis.

[42] Lum C, Ahmed ME, Patro S, Thornhill R, Hogan M, Iancu D, Lesiuk H, Dos Santos M, Dowlatshahi D (2014). Computed tomographic angiography and cerebral blood volume can predict final infarct volume and outcome after recanalization. Stroke.

[43] Al-Buhairi AR, Phillips SJ, Llewellyn G, Jan MM (1998). Prediction of infarct topography using the Oxfordshire Community Stroke Project classification of stroke subtypes. J Stroke Cerebrovasc Dis.

[44] Mead GE, Lewis SC, Wardlaw JM, Dennis MS, Warlow CP (2000). How well does the Oxfordshire community stroke project classification predict the site and size of the infarct on brain imaging?. J Neurol Neurosurg Psychiatry.

[45] Silvestrini M, Vernieri F, Pasqualetti P, Matteis M, Passarelli F, Troisi E, Caltagirone C (2000). Impaired cerebral vasoreactivity and risk of stroke in patients with asymptomatic carotid artery stenosis. JAMA.

[46] Zaidi SF, Aghaebrahim A, Urra X, Jumaa MA, Jankowitz B, Hammer M, Nogueira R, Horowitz M, Reddy V, Jovin TG (2012). Final infarct volume is a stronger predictor of outcome than recanalization in patients with proximal middle cerebral artery occlusion treated with endovascular therapy. Stroke.

[47] Yassi N, Churilov L, Campbell BC, Sharma G, Bammer R, Desmond PM, Parsons MW, Albers GW, Donnan GA, Davis SM (2015). The association between lesion location and functional outcome after ischemic stroke. Int J Stroke.

[48] Mohan KM, Wolfe CD, Rudd AG, Heuschmann PU, Kolominsky-Rabas PL, Grieve AP (2011). Risk and cumulative risk of stroke recurrence: a systematic review and meta-analysis. Stroke.

[49] Westendorp WF, Nederkoorn PJ, Vermeij JD, Dijkgraaf MG, van de Beek D (2011). Post-stroke infection: a systematic review and meta-analysis. BMC Neurol.

[50] Brott T, Marler JR, Olinger CP, Adams HP, Tomsick T, Barsan WG, Biller J, Eberle R, Hertzberg V, Walker M (1989). Measurements of acute cerebral infarction: lesion size by computed tomography. Stroke.

[51] Steffenhagen N, Campos CR, Poppe AY, Khan F, Kosior JC, Demchuk AM, Hill MD, Coutts SB (2010). Reliability of measuring lesion volumes in transient ischemic attack and minor stroke. Stroke.

