# Supplementary material for: CT angiography and CT perfusion improve prediction of infarct volume in patients with anterior circulation stroke
Source: Neuroradiology. 2016 Jan 14;58:327–37. doi: 10.1007/s00234-015-1636-z (PMC4819789; doi:10.1007/s00234-015-1636-z)
Supplement: Supplementary file 1 — (PDF 138 kb) [file 234_2015_1636_MOESM1_ESM.pdf]

SUPPLEMENTAL FILES

CT angiography and CT perfusion improve prediction of infarct volume in patients with anterior circulation stroke

Online Table 1. Multivariable analyses for prediction of infarct presence on follow-up imaging (n=906)

	**Model 1** Patient characteristics and non-contrast CT		**Model 2a** Addition of CT angiography		**Model 2b** Addition of CT perfusion		**Model 3** Addition of CT angiography and CT perfusion	
**Predictor**	Coefficient	OR (95% CI)[a]	Coefficient	OR (95% CI)[a]	Coefficient	OR (95% CI)[a]	Coefficient	OR (95% CI)[a]
Age (per decade)	-0.084	0.92 (0.81-1.04)	-0.113	0.89 (0.78-1.02)	-0.147	0.86 (0.75-0.99)*	-0.142	0.87 (0.76-1.00)*
Stroke severity (NIHSS)								
NIHSS 1-2	0.000 (ref)	1.00 (ref)	0.000 (ref)	1.00 (ref)	0.000 (ref)	1.00 (ref)	0.000 (ref)	1.00 (ref)
NIHSS 3-4	0.184	1.20 (0.76-1.89)	0.143	1.15 (0.72-1.85)	0.029	1.03 (0.63-1.68)	0.032	1.03 (0.63-1.69)
NIHSS 5-7	0.731	2.08 (1.34-3.22)**	0.648	1.91 (1.22-3.01)**	0.360	1.43 (0.89-2.31)	0.346	1.41 (0.87-2.29)
NIHSS 8-13	1.523	4.59 (2.60-8.10)***	1.225	3.40 (1.88-6.16)***	0.977	2.66 (1.43-4.93)**	0.924	2.52 (1.34-4.72)**
NIHSS >13	2.106	8.21 (3.73-18.08)***	1.274	3.57 (1.50-8.51)**	0.743	2.10 (0.82-5.37)	0.636	1.89 (0.71-4.99)
Time from symptom onset to scan (per hour)	0.050	1.05 (0.95-1.16)	0.047	1.05 (0.95-1.16)	0.100	1.11 (0.99-1.23)	0.092	1.10 (0.98-1.22)
Admission glucose level (per mmol/L)	0.037	1.04 (0.96-1.12)	0.037	1.04 (0.96-1.12)	0.043	1.04 (0.97-1.13)	0.043	1.04 (0.96-1.13)
IV-rtPA, IAT, or mechanical thrombectomy	-0.264	0.77 (0.51-1.15)	-0.323	0.72 (0.48-1.09)	-0.212	0.81 (0.52-1.25)	-0.215	0.81 (0.52-1.25)
**Non-contrast CT predictors**								
Hyperdense vessel sign	1.747	5.74 (2.55-12.92)***	1.196	3.31 (1.41-7.78)**	0.844	2.33 (0.91-5.94)	0.789	2.20 (0.85-5.74)
Non-contrast CT ASPECTS (per point decrease)	1.897	6.67 (2.71-16.40)***	1.323	3.75 (1.51-9.32)**	1.119	3.06 (1.28-7.32)*	1.047	2.85 (1.16-7.01)*
**CT angiography predictors**								
CT angiography source images ASPECTS (per point decrease)			0.529	1.70 (1.21-2.39)**			0.046	1.05 (0.74-1.49)
Proximal intracranial occlusion			0.885	2.42 (1.20-4.88)*			0.209	1.23 (0.54-2.80)
Poor collaterals			0.971	2.64 (0.83-8.39)			0.350	1.42 (0.39-5.17)
Significant ipsilateral carotid stenosis or occlusion			0.429	1.54 (0.85-2.78)			-0.079	0.92 (0.46-1.87)
**CT perfusion predictors**								
Cerebral blood volume (CBV) ASPECTS (per point decrease)					0.885	2.42 (1.62-3.63)***	0.833	2.30 (1.53-3.46)***
Mean transit time (MTT) ASPECTS (per point decrease)					0.029	1.03 (0.88-1.20)	0.008	1.01 (0.85-1.20)
Penumbra area (per SD; 19.9 cm^2)					0.615	1.85 (1.12-3.06)*	0.605	1.83 (1.09-3.07)*
Infarct core area (per SD; 13.7 cm^2)					0.224	1.25 (0.54-2.91)	0.173	1.19 (0.50-2.85)
**Model performance**								
R^2	0.39		0.45		0.53		0.53	
AUC value	0.818	(0.792-0.845)	0.849	(0.825-0.873)	0.891	(0.870-0.912)	0.892	(0.871-0.913)

[a]Confidence intervals are calculated using the standard errors of the model coefficients before shrinkage.
*p<0.05; **p<0.01; ***p<0.001.

Online Table 2. Multivariable analyses for prediction of infarct volume on follow-up imaging (n=906)

Predictor	**Model 1** Patient characteristics and non-contrast CT			**Model 2a** Addition of CT angiography			**Model 2b** Addition of CT perfusion			**Model 3** Addition of CT angiography and CT perfusion		
	B	95% CI	Standardized B	B	95% CI	Standardized B	B	95% CI	Standardized B	B	95% CI	Standardized B
Age (per decade)	-3.01	-7.05-1.03	-0.04	-3.51	-7.12-0.09	-0.05	-3.34	-7.00-0.31	-0.05	-3.53	-7.02--0.05*	-0.06
Stroke severity (NIHSS)												
NIHSS 1-2	0.00 (ref)			0.00 (ref)			0.00 (ref)			0.00 (ref)		
NIHSS 3-4	2.26	-16.87-21.40	0.03	2.79	-14.38-19.96	0.04	-1.06	-18.37-16.24	-0.02	0.90	-15.64-17.45	0.01
NIHSS 5-7	25.54	8.21-42.87**	0.34	24.50	8.90-40.10**	0.37	14.41	-1.35-30.16	0.22	18.65	3.57-33.74*	0.30
NIHSS 8-13	54.76	36.28-73.23***	0.74	39.89	23.07-56.70***	0.61	25.10	7.83-42.38**	0.38	27.17	10.64-43.70**	0.43
NIHSS >13	100.50	81.33-119.67***	1.36	68.19	49.92-86.46***	1.04	52.01	33.30-70.72***	0.79	51.83	33.71-69.95***	0.82
Time from symptom onset to scan (per hour)	-0.68	-4.02-2.66	-0.01	-0.62	-3.59-2.35	-0.01	0.05	-2.95-3.06	0.001	-0.04	-2.91-2.82	-0.001
Admission glucose level (per mmol/L)	3.04	0.66-5.43*	0.04	2.34	0.19-4.49*	0.04	2.93	0.77-5.09**	0.04	2.45	0.38-4.53*	0.04
IV-rtPA, IAT, or mechanical thrombectomy	-10.66	-25.18-3.86	-0.14	-14.14	-27.13--1.14*	-0.21	-10.64	-23.75-2.48	-0.16	-12.23	-24.79-0.34	-0.19
**Non-contrast CT predictors**												
Hyperdense vessel sign	33.90	19.87-47.93***	0.46	13.97	0.33-27.60*	0.21	5.60	-7.92-19.12	0.08	5.26	-8.06-18.57	0.08
Non-contrast CT ASPECTS (per point decrease)	22.09	18.35-25.82***	0.30	11.20	7.46-14.94***	0.17	13.81	10.26-17.36***	0.21	9.39	5.76-13.01***	0.15
**CT angiography predictors**												
CT angiography source images ASPECTS (per point decrease)				11.75	8.91-14.58***	0.18				7.44	4.52-10.35***	0.12
Proximal intracranial occlusion				2.93	-10.71-16.58	0.04				-11.21	-25.85-3.44	-0.18
Poor collaterals				49.67	34.61-64.74***	0.75				32.94	17.84-48.04***	0.52
Significant ipsilateral carotid stenosis or occlusion				16.03	3.05-29.02*	0.24				15.24	2.21-28.28*	0.24
**CT perfusion predictors**												
Cerebral blood volume (CBV) ASPECTS (per point decrease)							6.58	2.54-10.62**	0.10	4.97	1.05-8.89*	0.08
Mean transit time (MTT) ASPECTS (per point decrease)							1.94	-1.20-5.09	0.03	1.07	-2.12-4.27	0.02
Penumbra area (per SD; 19.9 cm^2)							3.12	-4.67-10.91	0.05	1.93	-5.51-9.36	0.03
Infarct core area (per SD; 13.7 cm^2)							24.11	15.70-32.51***	0.36	16.81	8.47-25.14***	0.27
**Model performance**												
R^2	0.44			0.55			0.54			0.58		
Sigma[a]	74.06			65.81			66.21			63.12		

[a]Sigma is the estimated standard error of the Tobit regression and is comparable with the root mean squared error in linear regression.
*p<0.05; **p<0.01; ***p<0.001

Online Table 3. Prediction of infarct presence and infarct volume (Excel file)

Please see the separate Excel file to use the interactive calculation sheet for prediction of infarct presence and infarct volume with patient characteristics, non-contrast CT, CT angiography and CT perfusion.
